# Supplementary figures and images for: Head capsule stacking by caterpillars: morphology complements behaviour to provide a novel defence
Source: PeerJ. 2016 Feb 29;4:e1714. doi: 10.7717/peerj.1714 (PMC4782731; doi:10.7717/peerj.1714)

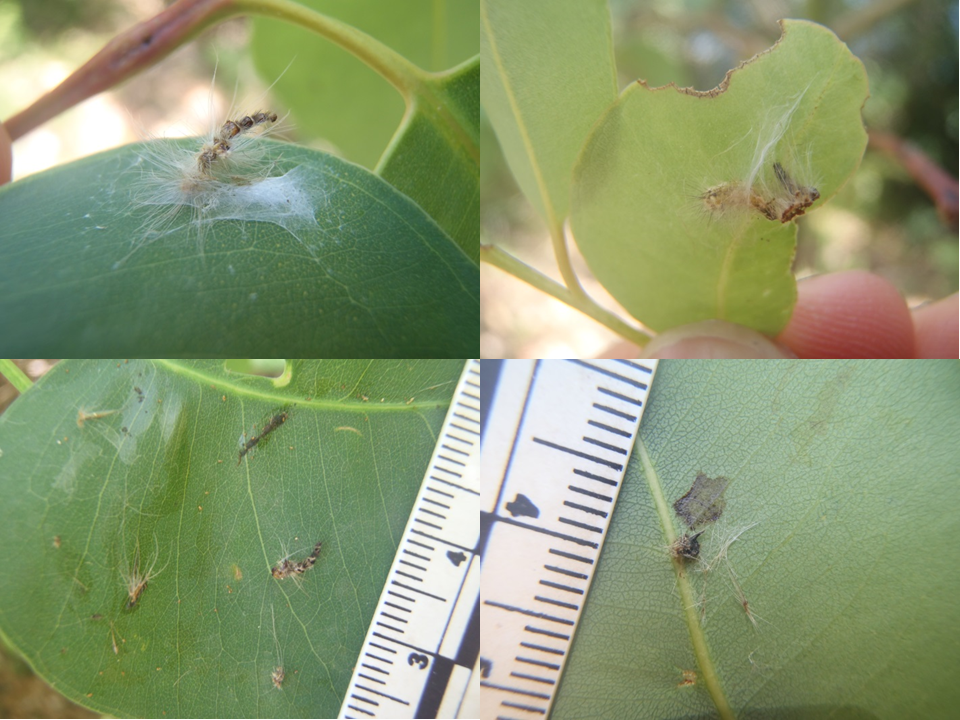

Supplement: Figure S1 — Examples of evidence of predation, likely from spiders, on caterpillars during the field experiment testing the influence of stacked head capsules on rates of predation. [file peerj-04-1714-s002.png]
