# Supplementary material for: Head capsule stacking by caterpillars: morphology complements behaviour to provide a novel defence
Source: PeerJ. 2016 Feb 29;4:e1714. doi: 10.7717/peerj.1714 (PMC4782731; doi:10.7717/peerj.1714)
Supplement: Video S1 — A video of an attack by the pentatomid bug on a Uraba lugens caterpillar, showing how it uses its head capsule stack to defend itself. [file peerj-04-1714-s003.docx]

This is a link to the Supplementary Video (which was too big to upload):

https://www.dropbox.com/s/559a9j23qdeu466/P3030055.MP4?dl=0
